# Supplementary figures and images for: PIKfyve/Fab1 is required for efficient V-ATPase and hydrolase delivery to phagosomes, phagosomal killing, and restriction of Legionella infection
Source: PLoS Pathog. 2019 Feb 7;15(2):e1007551. doi: 10.1371/journal.ppat.1007551 (PMC6382210; doi:10.1371/journal.ppat.1007551)

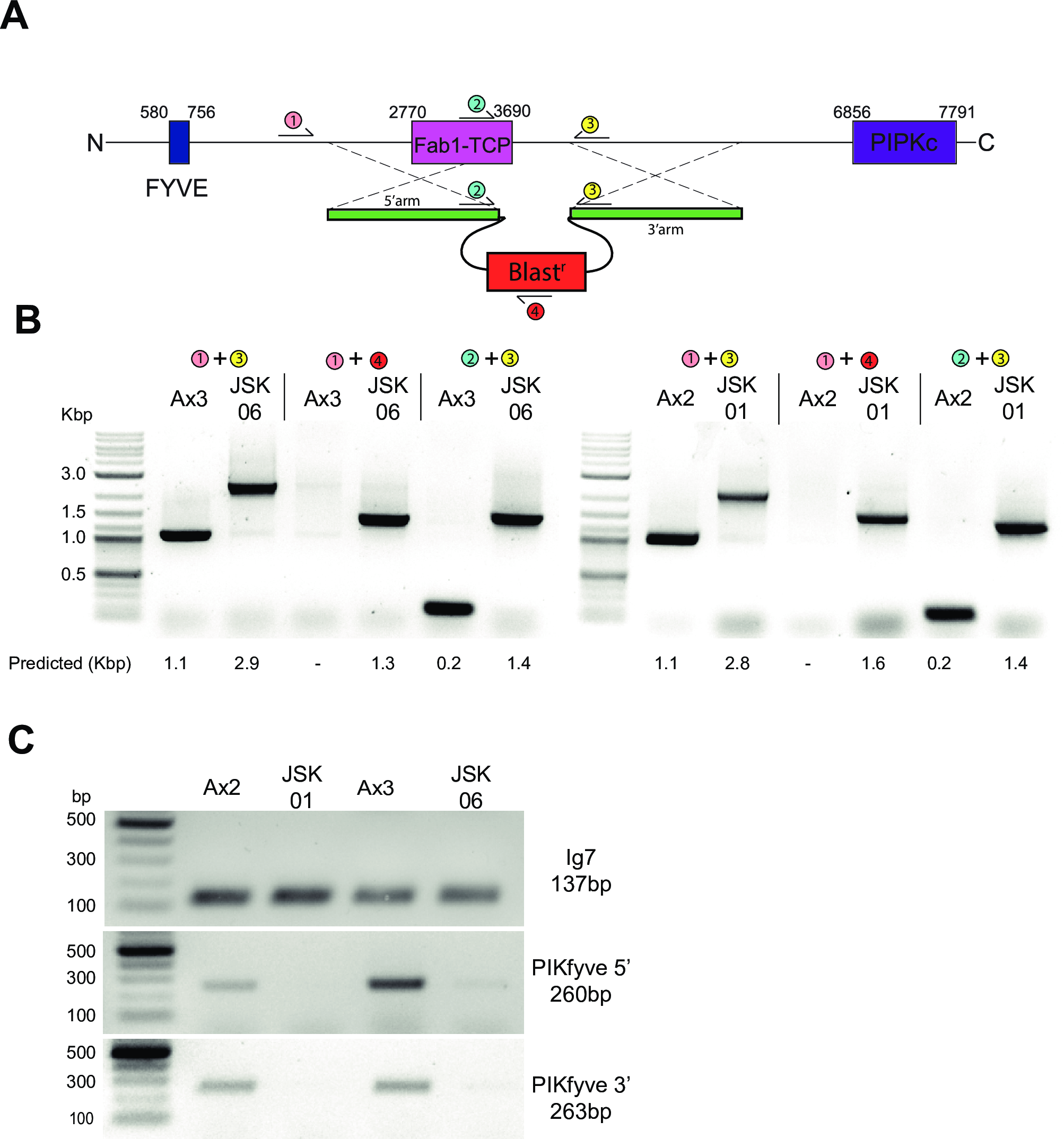

Supplement: S1 Fig — (A) Schematic representation of the Dictyostelium PIKfyve genomic locus indicating the homology arms, blasticidin resistance cassette. Primers used to screen the mutated locus are shown and labelled as circled numbers. (B) PCR screens of the genomic locus for strains JSK06 (Ax3-derived PIKfyve- (1)) and the Ax2-derived mutant JSK01. Three primer combinations were used to verify correct recombination, and loss of the wild-type allele. (C) Confirmation of loss of PIKfyve mRNA by RT-PCR. The mitochondrial large subunit rRNA Ig7 was used as positive control, as well as primers to the gene either 5’ or 3’ to the blasticidin insertion site. Primer sequences used are listed in S1 Table. (TIF) [file ppat.1007551.s001.tif]

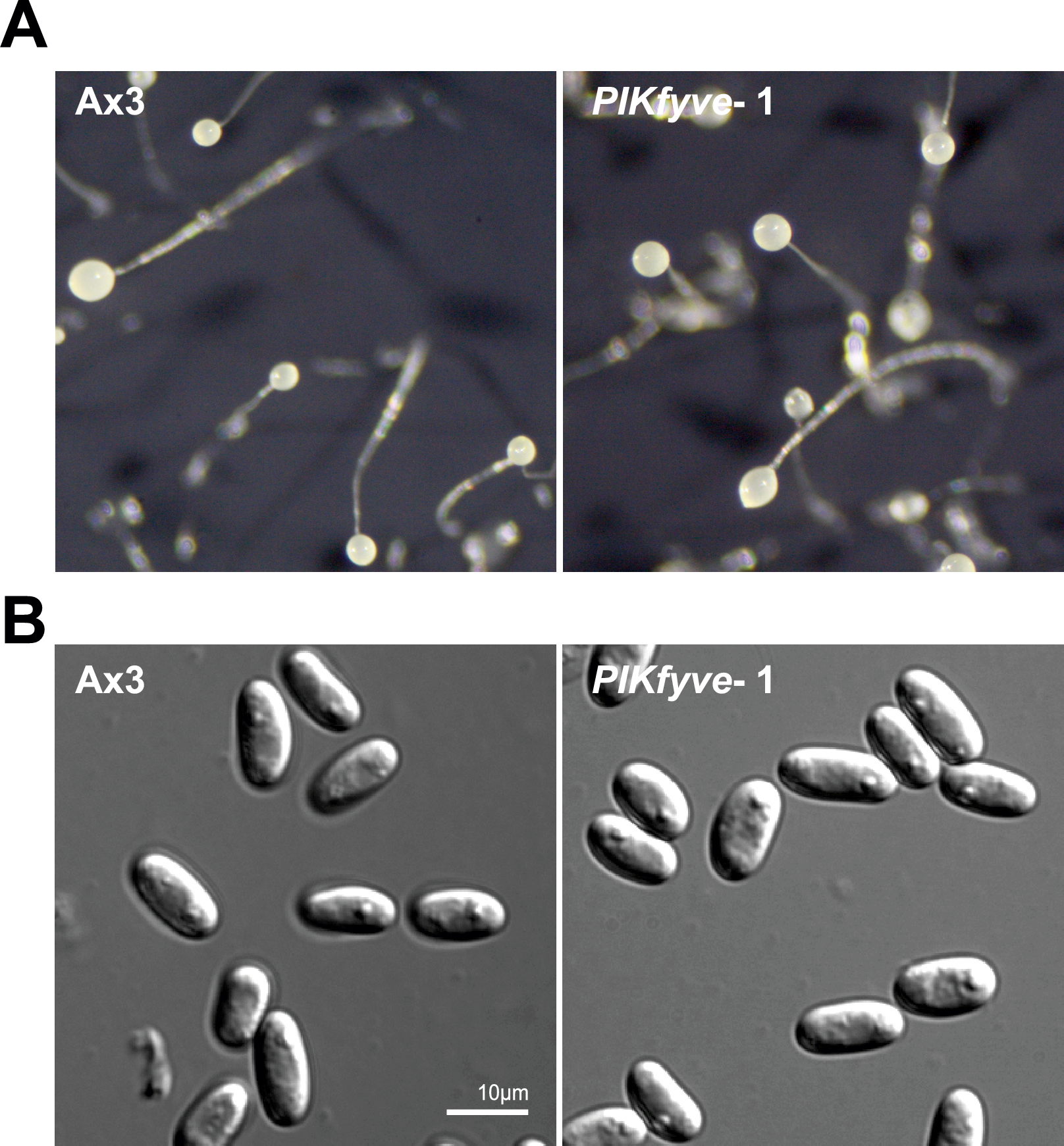

Supplement: S2 Fig — (A) Images of Dictyostelium fruiting bodies formed on filter discs, indicating a normal morphology and proportioning in the absence of PIKfyve. (B) Higher magnification differential interference contrast (DIC) images of pores collected from the fruiting bodies in (A). (TIF) [file ppat.1007551.s002.tif]

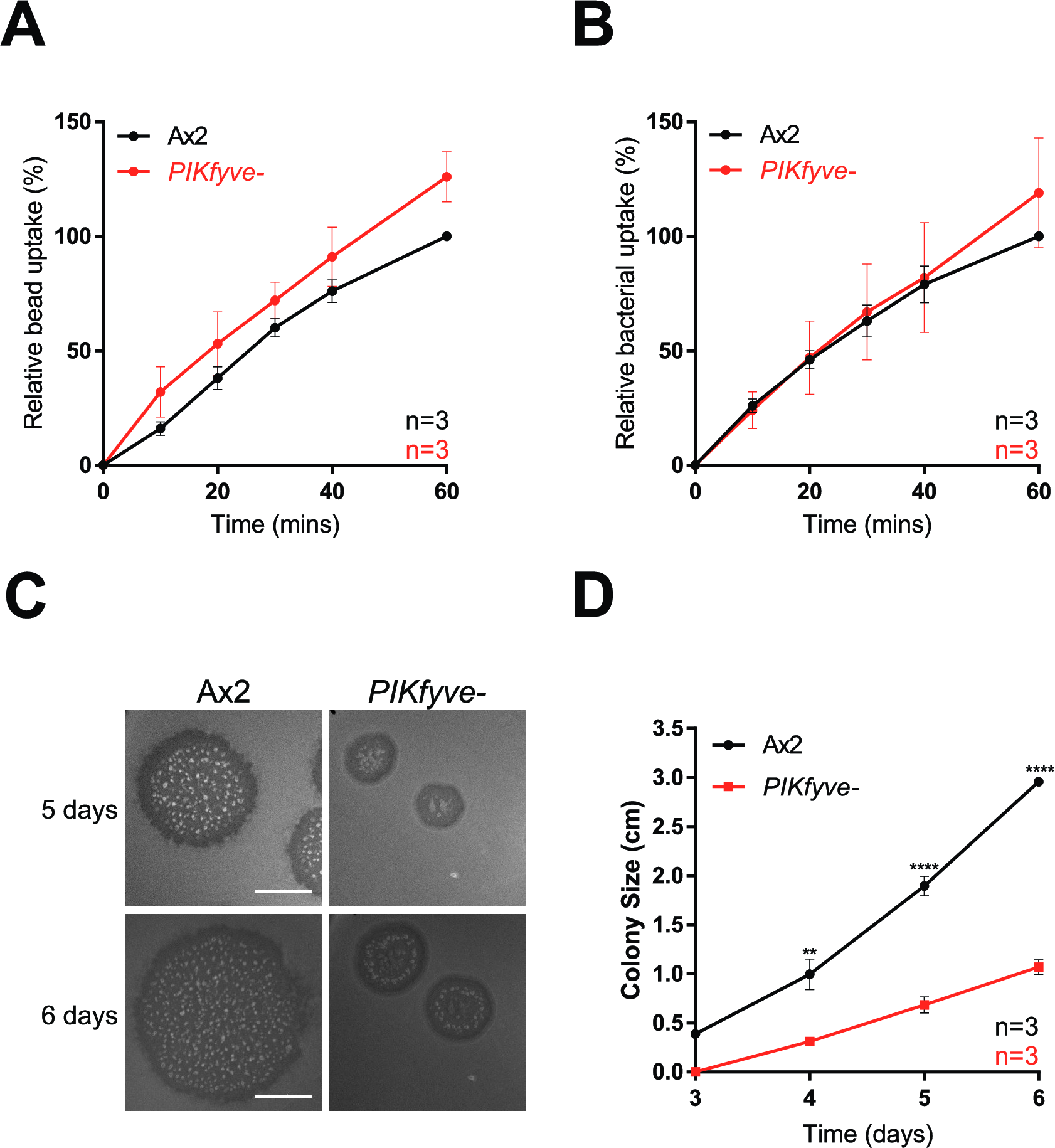

Supplement: S3 Fig — Phagocytosis of (A) of 1 μm beads or (B) GFP-expressing Mycobacterium smegmatis measured by flow cytometry, is normal in PIKfyve-null cells. (C) Growth on lawns of K. pneumoniae is impaired. Colony diameter over time is plotted in (D). All data are means +/- SD. (TIF) [file ppat.1007551.s003.tif]

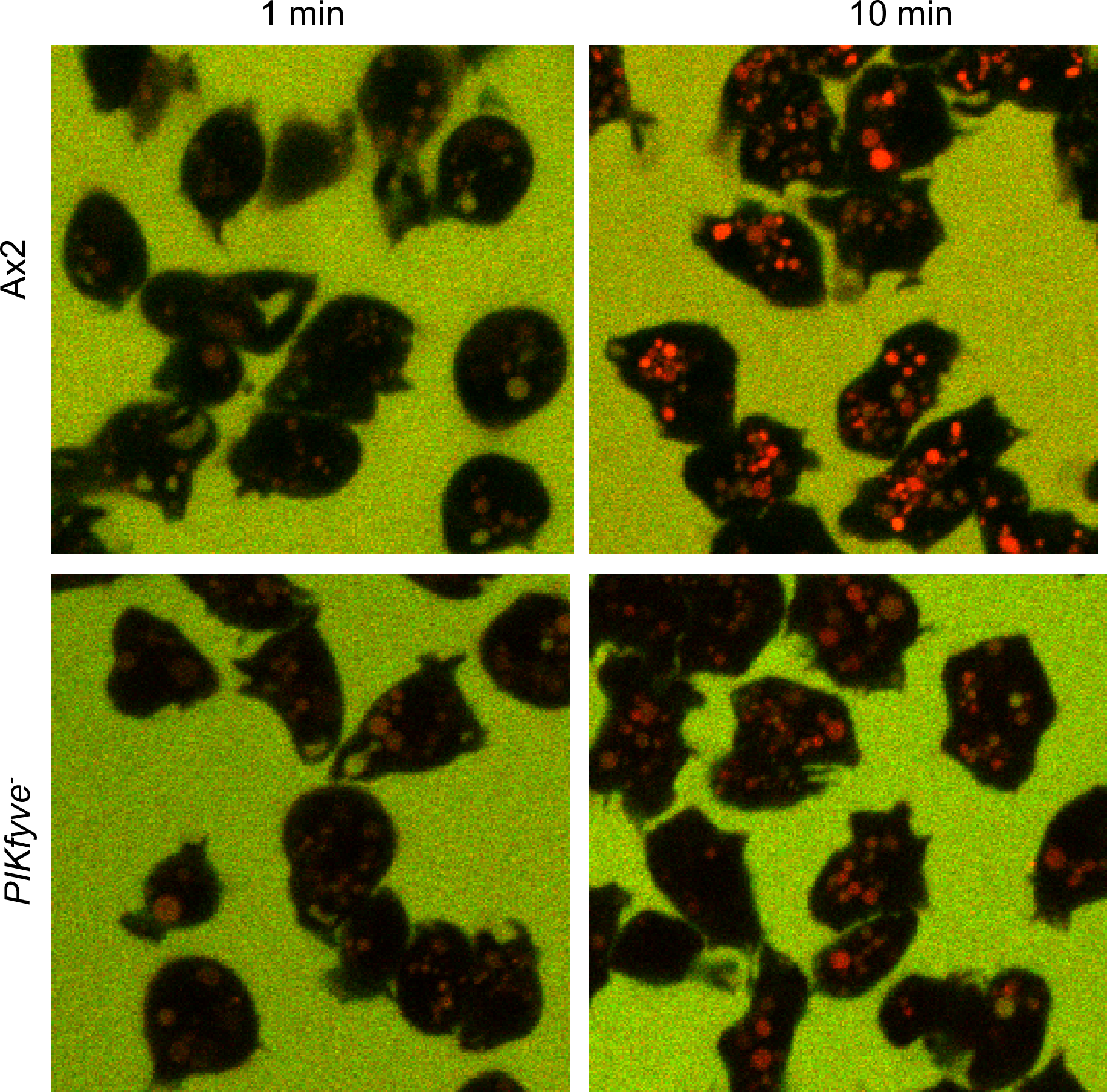

Supplement: S4 Fig — Cells were incubated in a mixture of 0.4 mg/ml FITC and 4 mg/ml TRITC dextran for the times indicated. Images were then captured on a confocal microscope. In this assay, vesicles of a neutral pH are yellow and become progressively more red as they acidify and FITC fluorescence is quenched. PIKfyve- cells remain able to acidify their macropinosomes within 10 minutes. (TIF) [file ppat.1007551.s004.tif]

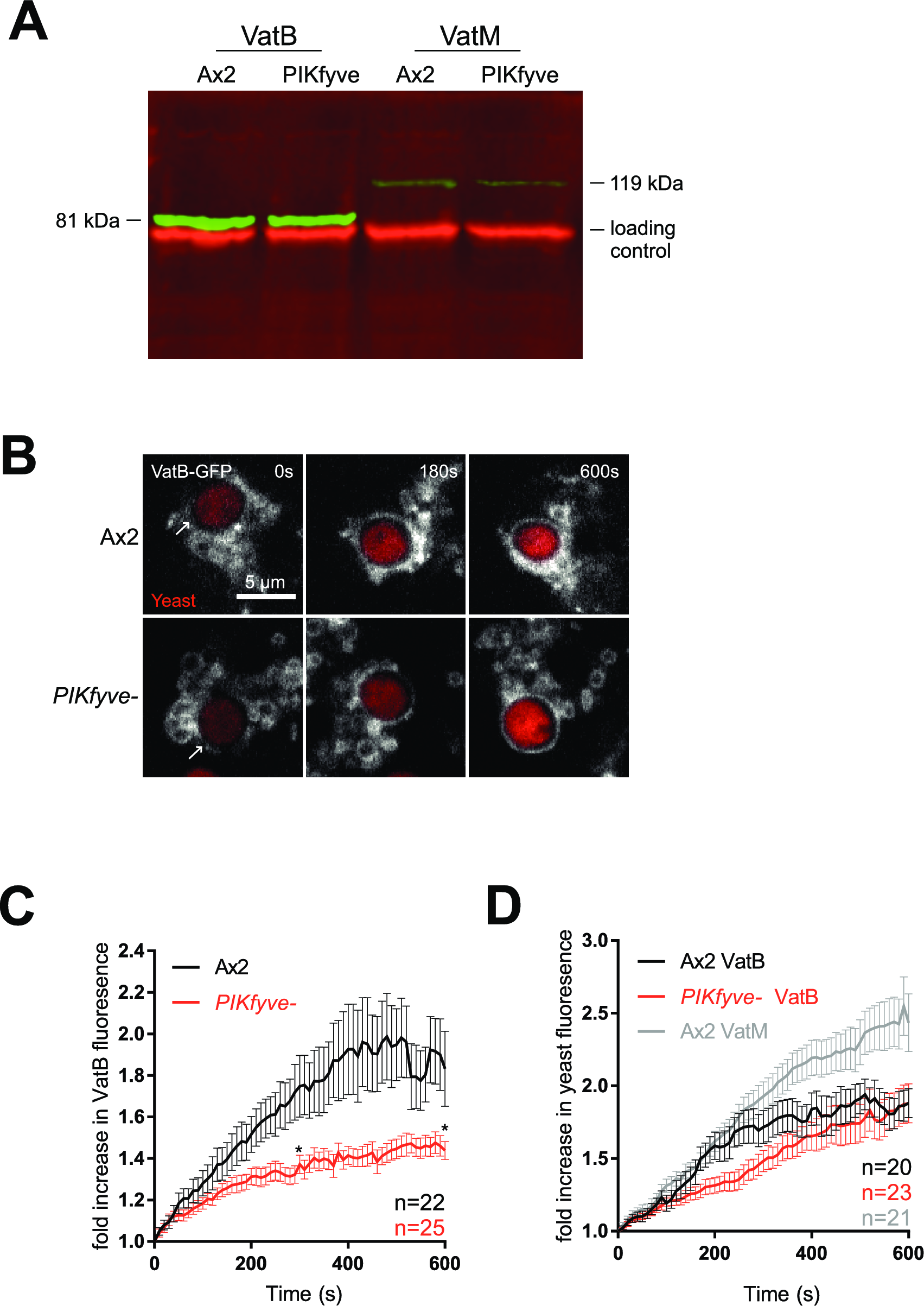

Supplement: S5 Fig — (A) Western blot of cells expressing VatB-GFP or GFP-VatM, probed with an anti-GFP antibody (green). There was no difference in expression levels between Ax2 and PIKfyve- cells for either reporter. However, VatB-GFP was expressed at higher levels than GFP-VatM, likely because it is present in 3 copies per V-ATPase complex. Loading control is the mitochondrial protein MCCC1, recognised by Alexa680-conjugated streptavidin (red). (B) Recruitment of VatB-GFP to phagosomes containing pHrodo-labelled yeast. (C) Automated image analysis of VatB-GFP recruitment as described in Fig 3, showing reduced recruitment in PIKfyve-null cells. (D) Phagosome acidification, measured by the increase in pHrodo fluorescence over time. Note that expression of VatB-GFP in Ax2 cells significantly reduces phagosome acidification relative to GFP-VatM expressing cells, indicating disruption of V-ATPase activity. Values plotted are mean +/- SEM. (TIF) [file ppat.1007551.s005.tif]

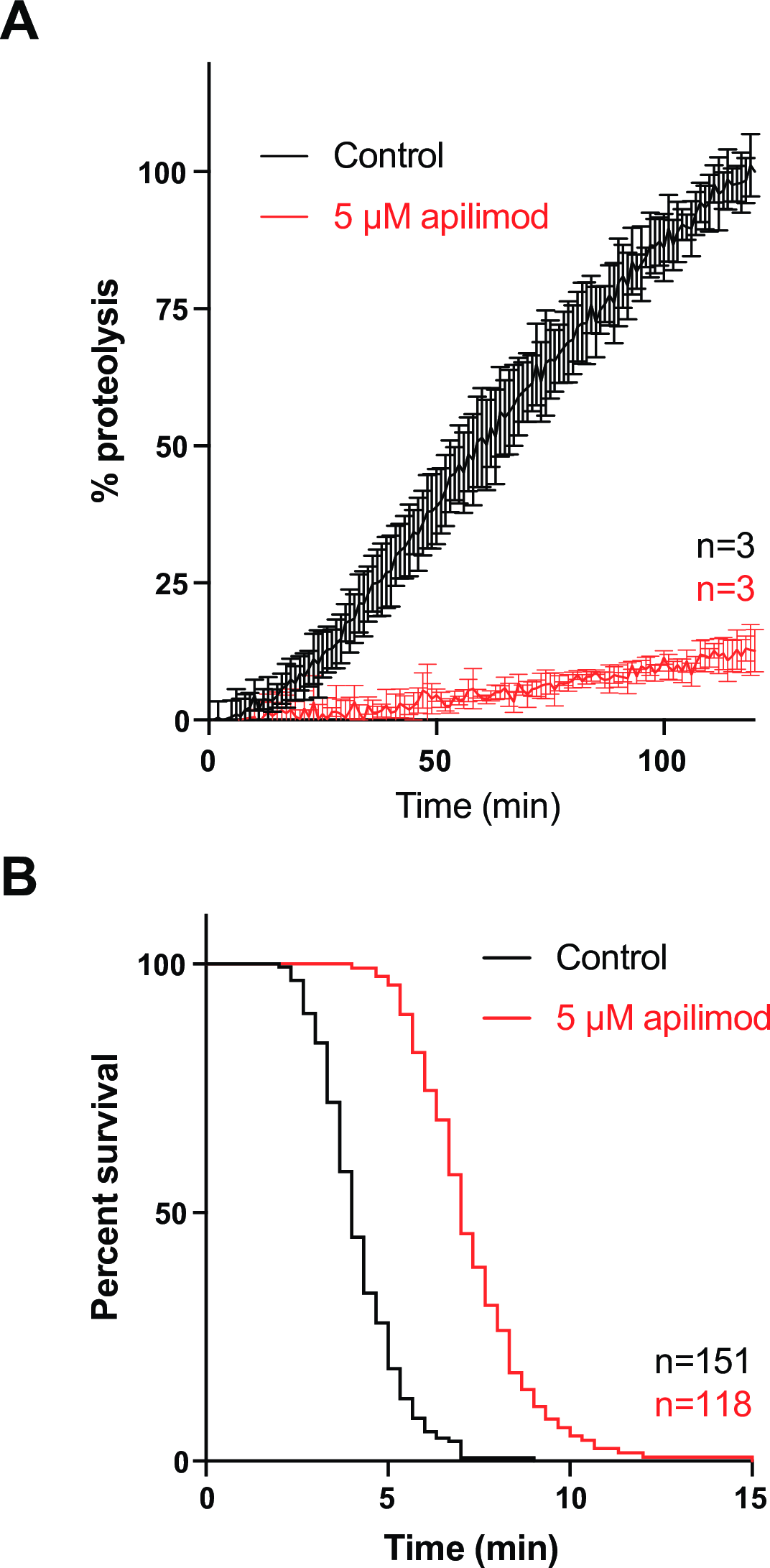

Supplement: S6 Fig — (A) Phagosomal proteolysis of Ax2 cells either untreated, or treated with 5 μM apilimod. As defects are in hydrolase delivery rather than activity, cells were pre-treated for 2 hours prior to the experiment. (B) Intracellular survival of GFP-expressing Klebsiella pneumoniae determined by the time taken for GFP fluorescence to be quenched post-phagocytosis. (TIF) [file ppat.1007551.s006.tif]
