# Supplementary material for: PIKfyve/Fab1 is required for efficient V-ATPase and hydrolase delivery to phagosomes, phagosomal killing, and restriction of Legionella infection
Source: PLoS Pathog. 2019 Feb 7;15(2):e1007551. doi: 10.1371/journal.ppat.1007551 (PMC6382210; doi:10.1371/journal.ppat.1007551)
Supplement: S1 Table — (DOCX) [file ppat.1007551.s007.docx]

| Use | Name/number | Sequence |
| --- | --- | --- |
| gDNA screen | 1 | GGTATTTCTTTAGCATTAAATGTAAAACC |
|  | 2 | AATGATTTAACATTGGGTGAATTCCTTAG |
|  | 3 | CCAGCACGCTGTACTGGC |
|  | 4 | TTCGGCAGTACATATTGAAGCG |
| RT-PCR | 5’arm fw | GGGTTGGATCAAAGTCAAATGAAAG |
|  | 5’arm rv | CCTATTGCCATTGCCATTGC |
|  | 3’arm fw | TTCGCTGTTGGTGGACTTGT |
|  | 3’arm rv | AGAGATAAGGCATGGTTGGACC |
|  | Ig7 fw | TCCAAGAGGAAGAGGAGAACTGC |
|  | Ig7 rv | TGGGGAGGTCGTTACACCATTC |

**Supplementary table 1: Primers used to for screening and validating *PIKfyve* gene disruption**
